# Why Selective Publication of Statistically Significant Results Can Be Effective

**DOI:** 10.1371/journal.pone.0066463

**Published:** 2013-06-20

**Authors:** Joost de Winter, Riender Happee

**Affiliations:** Department of BioMechanical Engineering, Delft University of Technology, Delft, The Netherlands; State University of New York, Oswego, United States of America

## Abstract

Concerns exist within the medical and psychological sciences that many published research findings are not replicable. Guidelines accordingly recommend that the file drawer effect should be eliminated and that statistical significance should not be a criterion in the decision to submit and publish scientific results. By means of a simulation study, we show that selectively publishing effects that differ significantly from the cumulative meta-analytic effect evokes the Proteus phenomenon of poorly replicable and alternating findings. However, the simulation also shows that the selective publication approach yields a scientific record that is content rich as compared to publishing everything, in the sense that fewer publications are needed for obtaining an accurate meta-analytic estimation of the true effect. We conclude that, under the assumption of self-correcting science, the file drawer effect can be beneficial for the scientific collective.

## Replicability Crisis and the File Drawer Effect

It is widely held that “replicability of findings is at the heart of any empirical science” [1]. Replication is obtained if applying the same research design in an independent sample of participants yields the same result, meaning that any difference between the observed effect and the true (population) effect is insubstantial [1].

Concerns exist within the medical and psychological communities that many published findings are poorly replicable. Published research findings are often false positives [2] or gross exaggerations of the true effect [3], [4], especially in domains where effect sizes and sample sizes are small and the prior probability of a hypothesis being true is low [2], [5], [6]. According to Pashler and Harris [7], one can legitimately speak of a “replicability crisis”.

Poor replicability is, in part, caused by the file drawer effect, meaning that findings that are statistically significant are more likely to be submitted and accepted for publication than null results [8]–[12]. Selective reporting is typically regarded as a questionable research practice [13] and has been associated with researchers’ pressure on productivity and novelty [6], flexibility in data analysis [14], desire for media attention [15], aversion to null results [16], and with the fact that many journals have a low acceptance rate. As pointed out by Giner-Sorolla [17], a publication bottleneck exists because researchers carry out many studies while there are relatively few publication outlets. Young, Ioannidis, and Al-Ubaydli [18] similarly argued that journals create an artificial scarcity of publication opportunity and an illusion of exclusivity.

Many authors recommend that the file drawer effect should be eliminated and that *p* values and effect sizes should not be a criterion in the decision to submit and publish scientific work [1], [11], [16], [19]–[22]. Davison and Nevin [23], for example, recommend that editors and reviewers should not be biased towards publishing novel or different results, but should publish also null results. Ioannidis [24] envisions a future ideal in which we publish everything to make “the scientific record complete rather than fragmented and opportunistic”. This publication philosophy is also adopted by the journal PLoS One, which states it will publish all papers that are judged to be technically sound [25].

The recommendation to publish both statistically significant and nonsignificant results is valid if the aim is to maximize the replicability of individual research studies. After all, according to the regression-toward-the-mean phenomenon, extreme variables tend to be closer to the true effect on a repeating measurement. However, we argue that this recommendation is less defensible from the perspective of the scientific collective. With a simulation, we show that selective publication eventually yields a more accurate estimate of the true effect than publishing everything.

## Assumptions of the Simulation Study

Our simulation study acts on the premise that science is self-correcting, and that what has previously been the alternative hypothesis becomes the null hypothesis which researchers try to refute. This premise is in line with Bronowski [26] who explained that “science is essentially a self-correcting activity … scientists are people who correct the picture of the moment with another one, as a natural evolution towards a ‘true’ picture of the world”. Specifically, we assume that researchers test their hypothesis with respect to the prevailing consensus as assessed by a cumulative meta-analysis of studies published on the same research question.

**Figure 1:**
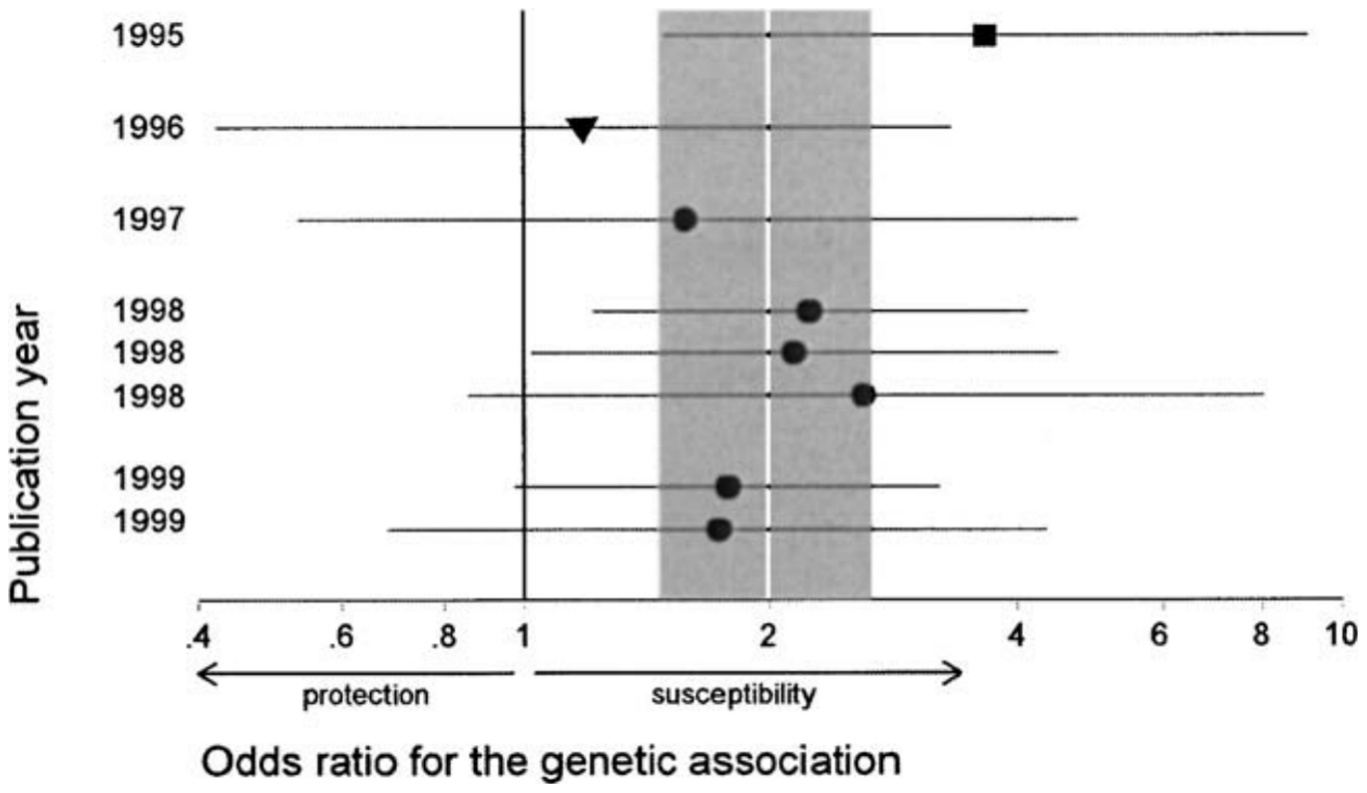
Illustration of Proteus phenemonen from Ioannidis and Trikalinos [27]. (Reprinted from Journal of Clinical Epidemiology, Vol. 58, J. P. Ioannidis and T. A. Trikalinos, Early extreme contradictory estimates may appear in published research: The Proteus phenomenon in molecular genetics research and randomized trials, pp. 543–549, 2005, with permission from Elsevier.) The figure shows odds ratios and 95% confidence intervals of “the relationship between the methylenetetrahydrofolate reductase (MTHFR) TT genotype in the mother and the risk of neural tube defects in the child”. The study with the strongest effect is shown by a square symbol and the study with the smallest effect is shown by a triangular symbol. The white line represents the summary odds ratio. The shaded area represents the 95% confidence interval of the summary odds ratio.

Ioannidis [2] argued that “negative” results become attractive for publication only if another researcher has published a “positive” result on the same research question. Elsewhere, Ioannidis and Trikalinos [27] coined the term “Proteus phenomenon” to describe their observation of “rapidly alternating extreme research claims and extremely opposite refutations” [2], particularly during the early accumulation of data. Figure 1 illustrates the Proteus phenomenon as observed in a genetic association study. It can be seen that the first publication substantially overestimates, and that the second publication underestimates the eventual summary effect. The Proteus phenomenon has previously been interpreted as an intricate form of publication bias [27]–[30]. We suggest that selective publication results in the Proteus phenomenon and contributes to an effective convergence towards the true effect in a cumulative meta-analysis.

Along with the self-correction assumption, our simulation assumes constant study quality and single hypothesis tests, each generating one *p* value. Of course, in reality, studies can be more complex and multiple hypotheses may be tested in a single assay. We do not contend reporting standards for complex research, such as making research data, protocols, and analytical codes publicly available (cf. [31]). Furthermore, the factor time is not included in our simulation models. That is, results are assessed per publication without taking into account study completion time and the time between study completion and publication. In reality, publication of research findings is not a sequential process as multiple researchers could be working on a topic in parallel.

## Simulation of the Publish Everything Approach versus the Selective Publication Approach

Computer simulations can be used to study dynamic processes of complex systems for which analytical solutions are not readily available. Herein, we use simulation to explore researchers’ publishing behavior as a function of previously published effects on the same research question. We compare two publication approaches: a Publish Everything Approach and a Selective Publication Approach. The prevailing opinion is that publishing everything is the preferred method and that selective reporting is a questionable research practice [13].

Suppose that researchers worldwide are investigating the strength of an effect by means of identical experiments and that the observed effects appear in published articles. The observed effects (*E_obs_*) are generated by independent random sampling of *n* subjects from a normal distribution with standard deviation of 1 and a mean *E_true_*.

In the Publish Everything Approach, observed effects are always published, irrespective of their magnitude or direction. In the Selective Publication Approach, statistically significant findings (*p* ≤ α) are published and nonsignificant findings (*p* > α) are not published (i.e., placed in the file drawer). The *p* value is determined using a two-tailed *z* test on *E_obs_* with respect to the null hypothesis *E_meta_*, which is the cumulative meta-analytic effect aggregating studies published on the same hypothesis so far, as in Eq. 1. In other words, a publication occurs only if the observed result (*E_obs_*) differs statistically significantly from the prevailing consensus (*E_meta_*).
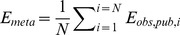
(1)


*E_obs,pub,i_* is the observed effect as published in the *i*-th publication and *N* is the number of publications so far. *E_meta_* is assumed to be 0 if no studies have been published yet.

We used the following input to the simulation: α = 0.05 (the false positive rate or significance level), *E_true_* = 0.3 (a relatively small true effect), and *n* = 50 (the sample size for each study). The simulation stopped when 40 studies were published in the Selective Publication Approach. The simulation was repeated 5,000 times, to be able to calculate the expected values of *E_obs,pub_* and *E_meta_*.

**Figure 2:**
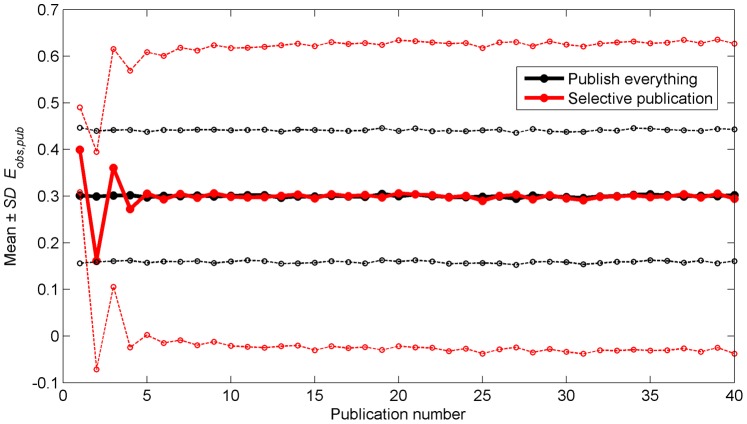
Mean (thicker solid lines) and mean plus/minus one standard deviation (thinner dashed lines) of observed effects of individual studies (*E_obs,pub_*) as a function of the number of publications. The means and standard deviations are calculated across the 5,000 repetitions of the simulation.

The mean observed effect of the published studies as a function of the publication number in Figure 2 shows an oscillating pattern for the Selective Publication Approach, akin to the Proteus phenomenon in Figure 1. The standard deviations around *E_true_* illustrate that published effects in the Selective Publication Approach differ more from the true effect than in the Publish Everything Approach. The high standard deviations are caused by the fact that, in the Selective Publication Approach, observed effects are published only if they differ more than 0.277 from *E_meta_*. Summarizing, replicability for the Selective Publication Approach is low, as demonstrated by the over- and underestimation of the true effect for the early publications, and by the large variability of published effects around *E_true_*.

**Figure 3:**
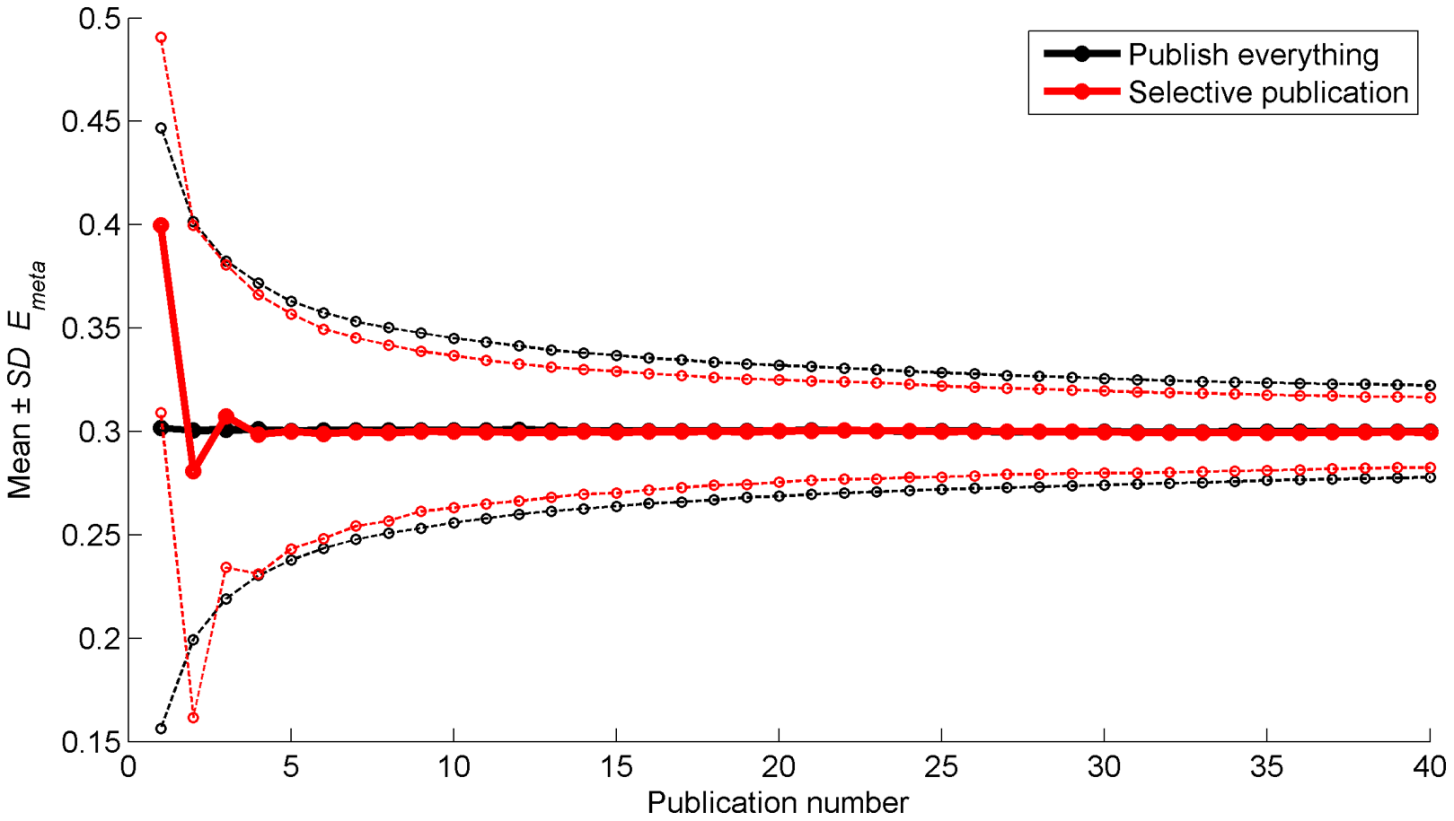
Mean (thicker solid lines) and mean plus/minus one standard deviation (thinner dashed lines) of the cumulative meta-analytic effect (*E_meta_*) as a function of the number of publications. The means and standard deviations are calculated across the 5,000 repetitions of the simulation.

Figure 3 shows that with the Selective Publication Approach, *E_meta_* values based on initial publications are on average biased with respect to *E_true_*. *E_meta_* converges towards *E_true_* after a few publications, indicating that this bias is rapidly nullified. The standard deviations in Figure 3 illustrate that *E_meta_* is on average closer to *E_true_* in the Selective Publication Approach than in the Publish Everything Approach. At the 40th publication, the *SD* of *E_meta_* for the Publish Everything Approach is 0.0222 and the *SD* for the Selective Publication Approach is 0.0170. For the Publish Everything Approach the *SD* value of 0.0170 is reached at the 68th publication.

The results in Figures 2 and 3 are in agreement with Ioannidis [32] who stated that “in some fields of research, we may observe diminishing effects for the strength of research findings and rapid alternations of exaggerated claims and extreme contradictions” (see also [33]). The decreasing support of a scientific claim over time is more commonly known as the decline effect [34].

**Figure 4:**
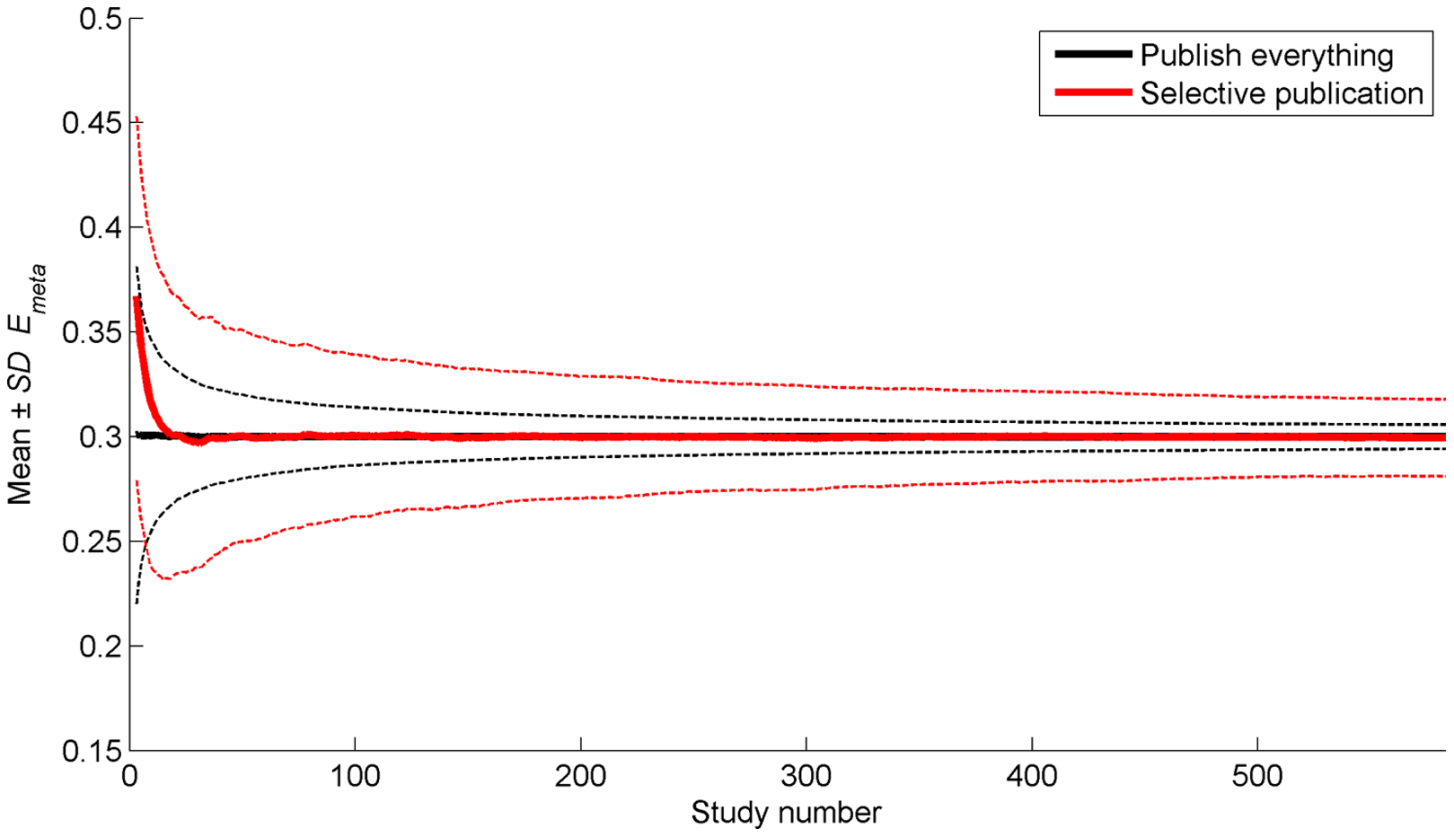
Mean (thicker solid lines) and mean plus/minus one standard deviation (thinner dashed lines) of the cumulative meta-analytic effect as a function of the number of studies. Note that the number of studies can vary per repetition because the simulation was terminated when 40 publications were done under the Selective Publication Approach. Only studies having more than 4,500 out of 5,000 *E_meta_* values available are shown (i.e., study numbers 3–585). The means and standard deviations are calculated across the repetitions of the simulation.

Figure 4 shows the same results as Figure 3, but now as a function of the number of studies instead of the number of publications. The standard deviations around *E_true_* indicate that *E_meta_* approximates *E_true_* more closely for the Publish Everything Approach. That is, when the results are assessed per study instead of per publication, the Publish Everything Approach performs more favorably than the Selective Publication Approach.

**Figure 5:**
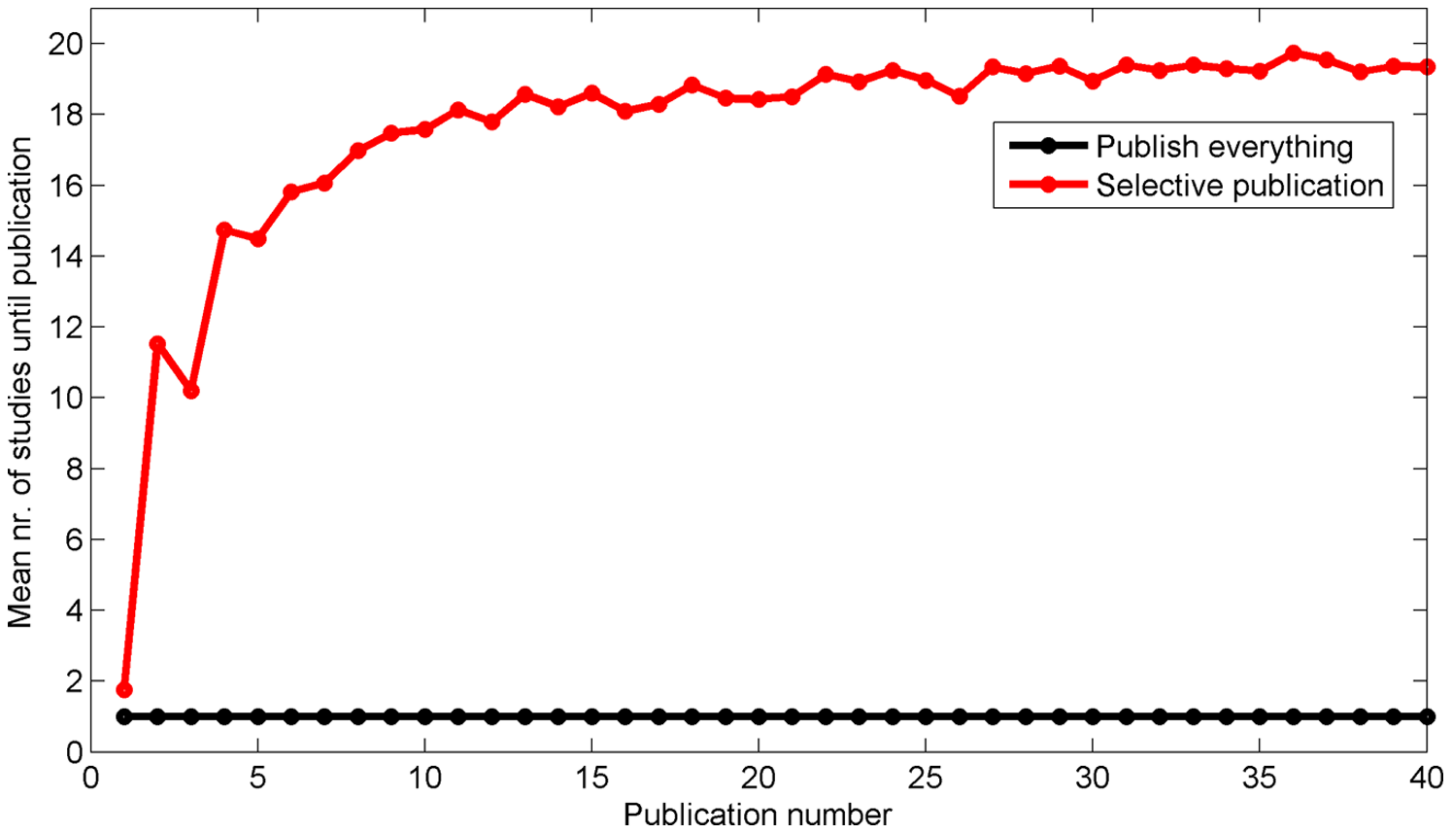
Mean number of studies until publication as a function of the number of publications. The means are calculated across the 5,000 repetitions of the simulation.

The mean number of studies until publication can be seen in Figure 5. In the Publish Everything Approach, this value equals 1 because each study is published. For the Selective Publication Approach, the probability of publication decreases with publication number, that is, when consensus establishes. The value converges to 20 (i.e., 1/α), meaning that the literature eventually grows 20 times as fast when publishing everything as compared to the Selective Publication Approach. The number of publications for the Publish Everything Approach is on average 704 (*SD* = 112), whereas the number of publications for the Selective Publication Approach is 40 for each repetition. The corresponding *SD*s of *E_meta_* (i.e., after publishing on average 704 and 40 studies) are 0.0053 and 0.0170 for the Publish Everything Approach and Selective Publication Approach, respectively.

The simulation code is provided as Supporting Information S1 and may be used for testing the effect caused by altering the simulation parameters. For example, with a stronger true effect, *E_true_* = 1 instead of *E_true_* = 0.3, the statistical power for the first publication of the Selective Publication Approach becomes virtually 1, meaning that the first study is always published. Accordingly, the over- and underestimation pattern does not occur, but the extreme opposite refutations and the comparative advantage of the Selective Publication Approach in terms of *SD* of *E_meta_* (cf. Figure 3) remain. In contrast, when using α = 0.01 instead of α = 0.05, statistical power decreases, and the systematic bias of *E_meta_* for the early publications has larger amplitude and takes more publications to fade out.

## Simulation of Inadequate Synthesis of the Literature

**Figure 6:**
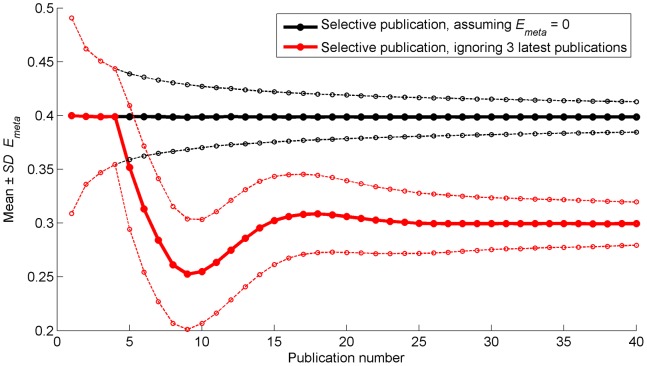
Mean (thicker solid lines) and mean plus/minus one standard deviation (thinner dashed lines) of cumulative meta-analytic effect as a function of the number of publications. The black lines represent the situation where *E_obs_* is tested with respect to 0. The red lines represent the situation when ignoring the 3 latest publications for determining *E_meta_*. The means and standard deviations are calculated across the 5,000 repetitions of the simulation.

In reality, researchers may not adequately synthesize the available literature. For example, researchers may not adapt their null hypothesis and simply continue to publish all results that differ statistically significantly from 0. Figure 6 illustrates that this would yield a systematic bias for the Selective Publication Approach; *E_meta_* is inflated, being about 0.10 greater than *E_true_* (0.3), and does not converge to *E_true_* as in Figure 3.

Another example of inadequate synthesis of the literature is ignoring published evidence. Figure 6 shows the effect of ignoring the 3 latest publications in the Selective Publication approach. The first 4 publications accumulate confidence in an exaggerated effect, and from the 5th publication results converge to *E_meta_* with a substantial delay and overshoot compared to the results in Figure 3. Our simulation ignored the 3 latest publications, which was considered a realistic situation. If a larger value than 3 is chosen, the period of the oscillation seen in Figure 6 will increase.

## Discussion

Our simulation study showed that instead of publishing everything, it is worthwhile to be selective and publish only research findings that are statistically significant. After a number of publications, selective publishing yields a more accurate meta-analytic estimation of the true effect than publishing everything (Figure 3). In other words, publishing nonreplicable results while placing null results in the file drawer can be beneficial for the scientific collective.

Our simulation assumed that science is self-correcting. That is, we assumed that researchers are committed to questioning and refuting previous publications. In some research fields, studies may be more likely to be published as long as the observed effect differs statistically significantly from 0, yielding a systematic bias of the cumulative meta-analytic effect (Figure 6). Another problem is that, in certain research fields such as social and behavioral sciences [35], replication studies may be unlikely and cumulative meta-analyses may never be done, resulting in unchallenged fallacies (cf. [36]). For example, if the true effect equals 0, the first selectively published effect will always deviate strongly from the true effect, and replication studies are required to refute this published claim. Because the self-correction assumption is probably untenable in many fields of science, we do not encourage selective publication. In line with this, we argue that the problem is not that researchers are averse to null results. The problem is the prejudiced researcher and the researcher who ignores or misrepresents previously published evidence on the same topic. Accordingly, efforts should go toward enhancing the self-correction mechanism and conducting a comprehensive literature synthesis prior to doing experiments.

According to our simulation, the Publish Everything Approach implies that content density of the literature database, defined as the information gained after synthesizing a given number of publications, will be suboptimal. Specifically, 68 publications were needed for the Publish Everything Approach for reaching the level of meta-analytic accuracy (i.e., *SD* of *E_meta_*) obtained after 40 publications in the Selective Publication Approach. Selective publishing yields a more accurate estimation of the true effect than publishing everything, as a function of the number of publications. However, publishing everything will yield a more accurate estimation than selective publishing, if taking into account all publications (cf. Figure 4). We argue that a given number of publications is the preferred criterion. Increasing the number of publications may place an unwanted burden on reviewers and editors, and we expect that no more than a fixed number of publications on a specific research question will be desired by a research community. This statement is in line with Nelson, Simmons, and Simonsohn [37] who argued that we should publish fewer papers in order to prevent what they called the “cluttered office effect”.

Science is becoming more competitive and researchers are pressured to publish frequently and in highly ranked journals, a phenomenon which has been associated with a rising prevalence of statistically significant effects in research journals [38]. We suggest that publication of significant effects, and the corresponding Proteus phenomenon, may in some cases be desirable or even optimal. Young, Ioannidis, and Al-Ubaydli [18] stated that we may have to “accept the current system as having evolved to be the optimal solution to complex and competing problems”. An analogy can be made with control theory, a discipline in the engineering sciences that deals with the behavior of dynamical systems and which is concerned with finding corrective actions that effectively reduce the sensed discrepancy between the system state and a reference value. Scientific discovery may be seen as an endeavor that minimizes the error between the prevailing opinion (*E_meta_*) and a reference value, the true effect (*E_true_*). Just like a person adjusting a shower spigot to reach a desired temperature (cf. [39]), researchers may publish their results in order to adjust a discrepancy between the prevailing consensus and the true effect. The strength of the corrective actions (cf. the amount of hot or cold water entering the shower) influences the rapidity with which errors are nullified, and is similar to the inverse of the α value used in the Selective Publication Approach. Selecting a low α results in a rapid response, but contributes to overshoot of the target value. A high α (e.g., α = 1; publishing everything) results in a sluggish response. This is qualitatively similar to adjusting the shower spigot with equal rapidity irrespective of the difference between the current temperature and the target temperature. Hence, it is legitimate to respond more strongly to effects that deviate more from the null hypothesis. As also pointed out by Drummond [40] and Fiedler et al. [41], being indifferent with respect to novelty or statistical significance is counterproductive.

## Supporting Information

Supporting Information S1 **Simulation code.**
(M) Click here for additional data file.
